# Melatonin and Selected Phytochemicals: A Promising Combination to Target Altered Metabolism in Cancer

**DOI:** 10.3390/nu18101515

**Published:** 2026-05-09

**Authors:** Wamidh H. Talib, Suha M. Sabri, Rawia M. Yousif, Rawan W. Hadi, Intisar Hadi AL-Yasari, SubbaRao V. Madhunapantula, Imre Soós, Douglas Law, Pongrác Ács

**Affiliations:** 1 Faculty of Allied Medical Sciences, Applied Science Private University, Amman 11931, Jordan; 2 Department of Clinical Nutrition and Dietetics, Applied Science Private University, Amman 11931, Jordan; s_sabri@asu.edu.jo (S.M.S.); rawanalyasari2001@gmail.com (R.W.H.); 3 Quality Control Department, Ministry of Trade, Baghdad 10001, Iraq; rawiaqcd@yahoo.com; 4 Department of Applied Biotechnology, College of Biotechnology, Al-Qasim Green University, Babylon 51013, Iraq; entesar@biotech.uoqasim.edu.iq; 5 Department of Biochemistry, Center of Excellence in Molecular Biology and Regenerative Medicine, JSS Medical College, JSS Academy of Higher Education & Research, Mysore 570015, Karnataka, India; mvsstsubbarao@jssuni.edu.in; 6 Faculty of Health Sciences, University of Pécs, H-7621 Pécs, Hungary; imre.soos@etk.pte.hu; 7 Department of Sport Sciences, Institute of Physiotherapy and Sport Sciences, University of Pécs, H-7621 Pécs, Hungary; 8 Faculty of Health and Life Sciences, Inti International University, Nilai 71800, Malaysia; douglas.law@newinti.edu.my; 9 Institute of Physiotherapy and Sports Science, Faculty of Health Sciences, University of Pécs, H-7621 Pécs, Hungary; pongrac.acs@etk.pte.hu; 10 Physical Activity Research Group, János Szentágothai Research Center, University of Pécs, H-7624 Pécs, Hungary

**Keywords:** Warburg effect, natural products, glycolysis, cancer, alternative therapies, bioactive compounds

## Abstract

Cancer is one of the leading causes of death globally. Conventional treatments such as surgery, radiotherapy, and chemotherapy are associated with several side effects, including chemoresistance and cancer relapse. As a result, there is a critical demand for new therapeutic agents to support the current therapeutic options. Recently, research started to focus on the possible positive interaction(s) between melatonin and many phytochemicals as a combination therapy to target altered metabolism in cancer. Such a combination has many advantages, including low toxicity, the possibility of having multiple synergistic anticancer effects, and low cost. This review comprehensively examines the current literature regarding combining melatonin with phytochemicals as a potential strategy to target cancer metabolism. It aims to contribute to the evolving landscape of cancer therapeutics by highlighting the potential benefits and future directions of melatonin–phytochemical combinations as an area of active research.

## 1. Introduction

Cancer is the second leading cause of death [1]. After cardiovascular diseases and is expected to cause a significant increase in the near future [2,3]. Due to the substantial strain that cancer and its therapies place on financial resources and healthcare systems, it is of high importance to prioritize the discovery of innovative preventative and therapeutic models that are not only cost-effective but also therapeutically efficient [4]. Surgery, radiotherapy, and chemotherapy are considered conventional anticancer approaches [5]. Nonetheless, the use of these approaches has not significantly improved the overall survival rate and is associated with several adverse effects such as cancer recurrence, drug resistance, vomiting, weight and hair loss, fatigue, and systemic toxicity that restrain their use [6,7]. Therefore, novel anticancer agents are required to complement existing cancer therapies [6,8].

Cancer metabolism refers to the remarkable ways in which cancer cells organize their metabolic processes to support their growth, survival, and proliferation. This is achieved by developing specialized strategies that enhance metabolic efficiency and promote survival in challenging environments [9]. Several approaches have been tested to target cancer metabolism, including the use of phytochemicals. Subsequently, phytochemicals have been used to target the protein, lipid, and carbohydrate metabolism of cancer cells. Generally, they act by starving the cancerous cells, hence disrupting their survival and proliferation. The ability of phytochemicals to target various crucial metabolic pathways makes them a valid alternative in treating cancer [10].

Phytochemicals are bioactive, non-nutritive substances found in plants that contribute to their flavor and color and are studied for their potential health benefits. These compounds exert their effects through multiple mechanisms, including acting as substrates for biochemical reactions, inhibitors of enzymatic activity, scavengers of reactive or toxic chemicals, and acting as cofactors of enzymatic reactions. A large body of evidence supports their role in the prevention of various diseases, including cancer. Phytochemicals have demonstrated anti-carcinogenic properties by reducing cell proliferation, inducing apoptosis, delaying metastasis, and inhibiting angiogenesis [6,11]. Moreover, they have been extensively studied as a potential resource for developing novel chemotherapies. Many anticancer agents registered from 1940 to 2014 originate from phytochemicals and showed high efficiency and selectivity [6].

Melatonin (*N*-acetyl-5-methoxy tryptamine) is a natural indole amine derived from tryptophan, exhibiting amphiphilic properties [12]. It is secreted by the pineal gland in mammals in response to darkness. However, melatonin can be found in other organs, including the retina, gastrointestinal tract (GIT), bone marrow, and lymphocytes [13]. The central circadian pacemaker in the paired suprachiasmatic nuclei (SCN) of the anterior hypothalamus triggers the nighttime pineal production of melatonin. Melatonin is a pleiotropic molecule with diverse biological activities including immunomodulation, antioxidative processes, and hematopoiesis [14,15]. In addition, several studies have reported the anticancer effects of melatonin, driven by different mechanisms of action [13,16].

Currently, the use of melatonin as an anticancer agent appears to be a promising and effective strategy for cancer management [17,18,19]. One of the newest fields involves its role in regulating various epigenetic processes, including histone modifications, biogenesis of ncRNAs, and modification of DNA methylation. Studies indicate that melatonin can modify the activity of enzymes like DNMTs, altering DNA methylation patterns, particularly in breast cancer cells [20].

Although the therapeutic potential of melatonin has been thoroughly examined, numerous studies have concentrated on its overall anticancer properties [21] and its role in regulating mitochondrial balance [22]. There is increasing interest in combining it with phytochemicals to improve effectiveness [16]. The previous literature has also laid the groundwork for targeting cancer metabolism through multi-targeted natural approaches [23] and specific signaling pathways [20]. However, this review specifically synthesizes the synergistic mechanisms of melatonin with selected plant-derived compounds in modulating the Warburg effect and lipid signaling, providing a more focused clinical and mechanistic perspective.

Cancer growth and progression occur through several mechanisms. Hence, it is thought that the efficacy of anticancer agents may be enhanced when multiple agents are combined [7,24]. This review summarizes recent findings on altered cancer metabolism and how melatonin, combined with phytochemicals, affects these metabolic pathways.

## 2. Methodology

The aim of this review is to identify relevant studies investigating the effects of melatonin and phytochemical compounds on cancer metabolism and their potential synergistic interactions. A comprehensive literature search was conducted using scientific databases, including PubMed and Google Scholar, with appropriate keywords related to the topic such as “melatonin,” “bioactive compounds,” “phytochemicals,” “cancer metabolism,” and “synergy.” Both in vitro and in vivo experimental studies, as well as mechanistic investigations and relevant review articles, were included. Studies focusing on non-cancer-related outcomes or lacking mechanistic relevance were excluded.

## 3. Altered Metabolism in Cancer

**Figure 1:**
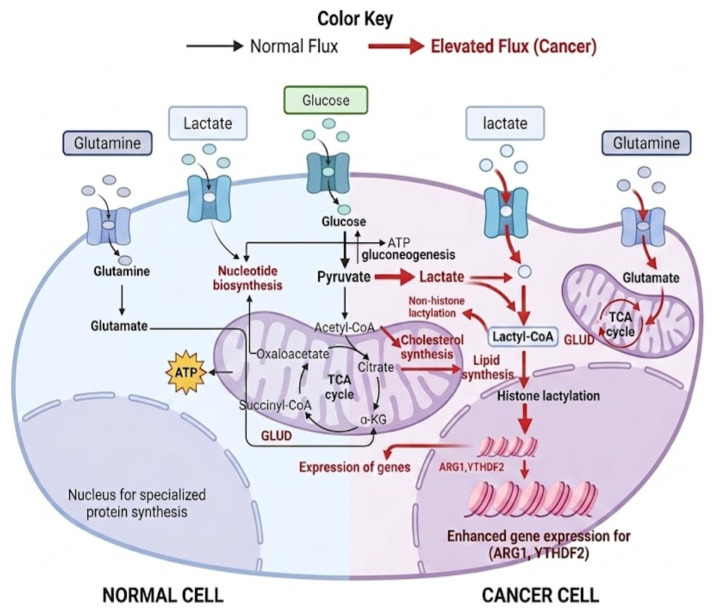
The altered metabolism in cancer cells resulted in an increase in the rate of metabolic pathways like glycolysis and the TCA cycle. The import of glucose, glutamine, and lactate is also accelerated. This mitochondrial dysfunction causes accumulation of intermediates that contribute to the main biosynthetic pathways including nucleotide, cholesterol, and lipid synthesis. Red color means higher metabolic activity.

Metabolic reprogramming is known to be a fundamental feature of cancer, allowing malignant cells to grow, proliferate, avoid programmed cell death, and adjust to various microenvironment stressors. Unlike normal cells, which depend on mitochondrial function and oxidative phosphorylation (OXPHOS) for energy production under aerobic conditions, cancer cells utilize glycolysis and lactate fermentation (Warburg effect) to boost energy production and provide intermediates for biosynthetic pathways [25]. Beyond glycolysis, tumors shift other metabolic programs such as lipid biosynthesis and glutaminolysis, both aiding biomass accumulation and maintaining redox hemostasis. These processes are often regulated by cancer signaling pathways alongside the inhibition of tumor suppression mechanisms (Figure 1) [26].

### 3.1. Glycolysis

The Warburg effect, first described by Otto Warburg in the 1920s, explains how cancer cells convert glucose to lactate despite the presence of oxygen. This metabolic shift allows tumor cells to rapidly meet their energy needs and provide essential building blocks, such as nucleotides, lipids, and amino acids that are necessary for cell proliferation and growth [27]. This alteration in metabolism is induced via major genetic changes that activate oncogenes such as *c-Myc* and *K-Ras*, and inhibit neoplasm suppressor genes, including *TP53*. These changes upregulate the glycolytic enzymes and transporters, such as pyruvate kinase M2 (PKM2), hexokinase 2 (HK2), lactate dehydrogenase A (LDHA), and monocarboxylate transporter 4 (MCT4), allowing tumors to grow despite unfavorable conditions that would normally cause apoptosis [28,29].

For instance, lactate dehydrogenase A (LDHA) converts glucose to lactate, which is then transported out of tumors by monocarboxylate transporter 4 (MCT4). As a result, the tumor microenvironment becomes acidic, which suppresses the immune response by inhibiting cytotoxic T cells, promoting immune evasion, and supporting immune-suppressive cells [30].

### 3.2. Mitochondrial Dysfunction and Oxidative Phosphorylation (OXPHOS)

**Figure 2:**
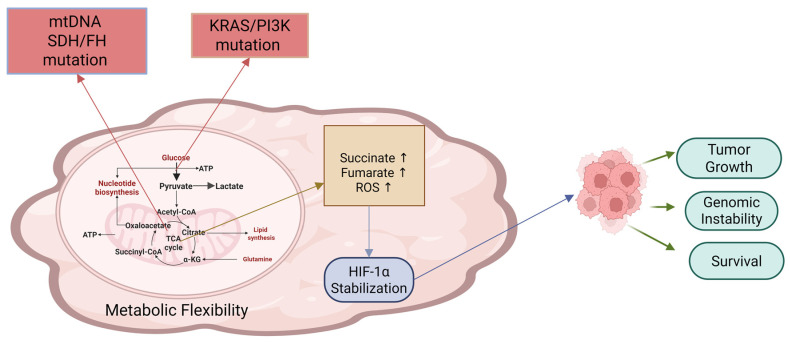
Cancer-associated metabolic flexibility is a direct result of mutation in mitochondrial DNA and some enzymes such as succinate dehydrogenase and fumarate hydratase. These modifications caused an increase in the concentration of succinate, fumarate, and reactive oxygen species. Accumulation of these metabolites results in HIF-1α stabilization which supports cancer survival. Arrows mean increased concentrations.

Various tumors maintain their dual metabolic activity, in which OXPHOS acts as a source of ATP when glycolysis becomes inadequate, helping tumors adapt to the harsh microenvironment caused by depletion of nutrients or oxygen. Mutations in mitochondrial DNA (mtDNA), some tumor-signaling molecules (e.g., *KRAS*, PI3K), and TCA cycle enzymes (e.g., SDH,FH) impair metabolic balance in normal cells and increase tumor-driving metabolites, such as fumarate, succinate and reactive oxygen species (ROS), all of which stabilize HIF-1α and promote tumor growth, survival and genomic instability [31] (Figure 2). For example, Acute Myeloid Leukemia (AML) displays a dual metabolism facilitating its resistance to therapy [26], making it a potential target where enhancement of OXPHOS could reverse chemoresistance [32].

### 3.3. Lipid Biosynthesis

**Figure 3:**
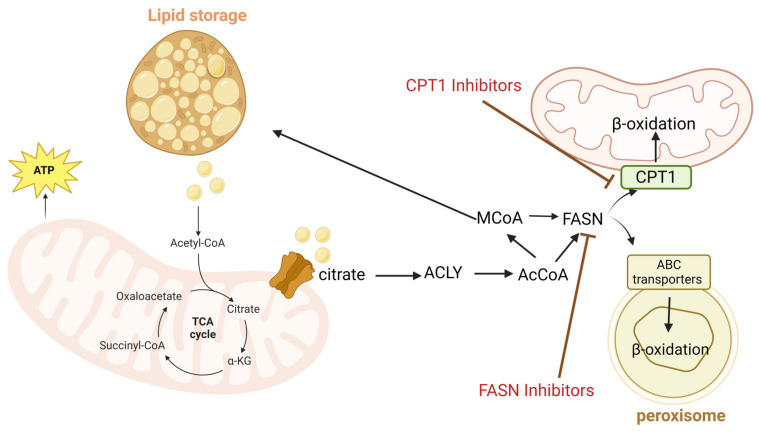
Lipid metabolism in cancer cells involves exporting citrate from mitochondria to the cytoplasm and production of Acetyl-CoA. Fatty acid synthase (FASN) catalyzes the production of fatty acids from Acetyl-CoA. Production of fatty acids support cancer cell survival by contributing to lipid storage and membrane biosynthesis. An arrow with line at the end means inhibition.

Lipids not only serve as membrane building blocks but also act as precursors for signaling molecules that support tumor survival and metastasis. Oncogenic signals such as PI3K, AKT, and mTOR enhance de novo lipogenesis (DNL) by upregulating key enzymes, such as fatty acid synthase, acetyl-CoA carboxylase (ACC), and ATP-citrate lyase (ACLY). This upregulation promotes membrane biogenesis, storage of energy, and increased lipid production in tumors [26] (Figure 3). The various mechanisms altering lipid metabolism promote fatty acid uptake, storage, and synthesis. For instance, triacylglycerol (TAG) and the enzyme acylglycerol-3-phosphate acyltransferase (AGPAT), which play essential roles in TAG biosynthesis, contribute to more aggressive cancer phenotypes [33].

In breast cancer, lipid metabolism differs based on subtypes. ER+ (Luminal) subtypes promote FASN and depend on de novo fatty acid synthesis, whereas basal-like/triple-negative tumors favor exogenous lipid uptake [34]. This makes enzymes such as FASN or ACLY appealing therapeutic targets, as their inhibition can impair tumor proliferation and increase chemosensitivity [35]. Carnitine palmitoyltransferase I (CPT1) is a key enzyme in the mitochondrial fatty acid oxidation pathway, and plays a critical role in providing an alternative ATP source and maintaining the rapid cell proliferation via nucleotide synthesis. For instance, the activation of the CPT1A enzyme in nasopharyngeal carcinoma enhances cell cycle progression by increasing nucleoside-driven metabolites, making this enzyme an important target to reduce disease progression and severity [36].

### 3.4. Glutaminolysis

Glutamine overconsumption is a hallmark of many cancers, including renal cell carcinoma, glioblastoma, and *Myc*-induced malignancies. Glutaminolysis converts glutamine to glutamate by glutaminase (GLS), followed by conversion to α-ketoglutarate (α-KG) through glutamine dehydrogenase (GDH) or aminotransferases (Figure 1) [37,38]. α-ketoglutarate enters the citric acid cycle (TCA), compensating resources such as carbon for anaplerotic flux and nitrogen for the synthesis of nucleotides [39]. Oncogenic signaling (e.g., via *Myc*) drives biosynthesis that creates a high demand for glutamine, and promotes the expression of transporters like SLC1A5. Inhibiting key transporters and enzymes, such as GLS and GDH, can deplete tumor cells. GLS inhibitors are under clinical investigation, making glutaminolysis a valuable target for therapeutic agents [40].

## 4. Melatonin Targeting Cancer Metabolism

### 4.1. Inhibition of Aerobic Glycolysis (Warburg Effect)

Melatonin (*N*-acetyl-5-methoxytryptamine) is an indoleamine that is produced endogenously, with diverse physiological roles in many malignancies. Melatonin inhibits glycolysis through the modulation of mitochondrial processes and metabolic mechanisms. For example, melatonin suppresses mTOR1, reducing mitochondrial respiration and inhibiting glycolysis through the downregulation of essential enzymes such as HK2 and LDHA as well as c-Myc protein [41]. Similarly, in head and neck squamous cell carcinoma, melatonin promotes metabolic shift toward oxidative phosphorylation, impedes glycolysis, and enhances reactive oxygen species production, resulting in apoptosis [42].

The reversal of aerobic glycolysis (Warburg effect) by melatonin has been demonstrated in several cancer cell types. Changes included reduced uptake of linoleic, decreased 13-HODE formation, suppressed DNA synthesis, and decreased phospho-activation of several key enzymes (Akt, GSK3β, ERK ½) [42,43].

**Figure 4:**
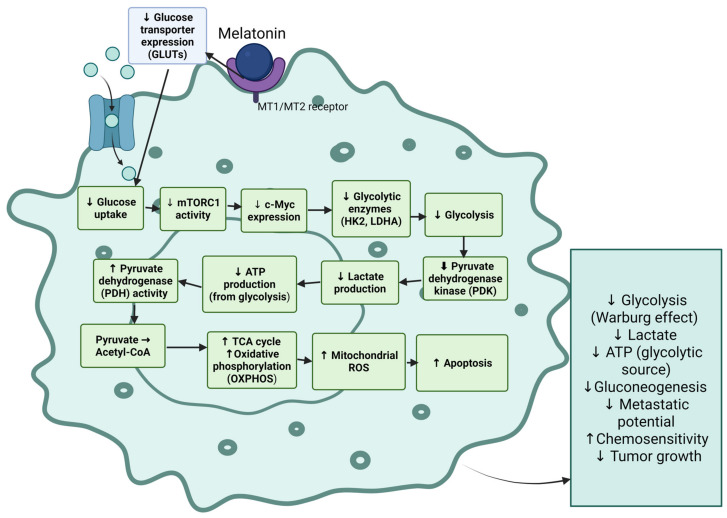
Melatonin alters cancer metabolism by targeting multiple metabolic intermediates. Melatonin causes a reduction in the expression of glucose transporters, inhibits hexokinase-2 and lactate dehydrogenase A, reduces expression of mTORC1 and *c-Myc*, and enhances pyruvate dehydrogenase activity. These changes decrease glycolysis and lactate production and enhance oxidative phosphorylation in cancer cells leading to apoptosis induction. An upward arrow means increased activity. A downward arrow means decreased activity.

Melatonin also regulates glucose transporter expression and the activities of enzymes involved in glycolysis and the pentose phosphate pathway [44]. In addition, it inhibits pyruvate dehydrogenase kinase and thereby enables the conversion of pyruvate into acetyl-CoA inside the mitochondria, facilitating oxidative phosphorylation and avoiding reliance on glycolysis [45,46] (Figure 4).

The increase in reactive oxygen species (ROS) is closely linked to oxidative stress, which plays a critical role in cancer progression. Under normal cellular conditions, there is a balance between ROS production and antioxidant defense systems; however, disruption of this balance leads to oxidative stress which can trigger mutagenesis and activate signaling pathways such as MAPK, ERK, and JNK. Melatonin acts as an antioxidant through both direct and indirect mechanisms. It directly scavenges free radicals, indirectly enhances the activity of antioxidant enzymes, and suppresses pro-oxidative enzymes [42,47].

Several studies have also shown that melatonin reduces tumor growth and glycolytic activity in various cancer types. In ovarian carcinoma, the treatment with melatonin reduced tumor growth volume by 50% in a nude mouse model. ENO1 has been identified as a melatonin-regulated protein that mediates downstream metabolic processes and is mainly involved in the glycolytic process of tumor growth [42,48].

By targeting glycolysis, melatonin decreases ATP production from this pathway and impairs other metabolic processes critical for cancer cell survival, such as gluconeogenesis and the tricarboxylic acid cycle [44,49]. This reprogramming of metabolism reduces tumor growth, diminishes metastatic potential, and enhances the sensitivity of cancer cells to chemotherapy [45,50].

### 4.2. Targeting Glutamine Metabolism

Dysregulation of glutamine metabolism, one of the important pathways supplying energy and growth to the cancer cell, is observed in various tumor cells. Cancer cells rely on glutamine uptake, which is essential for tumor growth and survival [46,51]. Alanine-serine-cysteine transporter 2 (ASCT2) is the primary glutamine transporter and is essential for the metabolic functions in cancer cells [52]. Glutamine also contributes to cellular antioxidant defense by promoting the production of GSH, which reduces ROS formation and oxidative stress [53]. Several studies showed increased expression of ASCT2 in tumor cells, which promotes the absorption of glutamine [18,54]. demonstrated that inhibiting ASCT2 significantly decreased glutamine production, α-KG production and ATP generation. Moreover, blocking glutamine uptake and therefore glutathione production through ASCT2 inhibition led to an increase in reactive oxygen species.

Melatonin had a significant antitumor effect by modulating various metabolic pathways, including glutamine metabolism. It influences critical metabolic pathways such as aerobic glycolysis, gluconeogenesis, and the TCA cycle that are of utmost importance regarding the survival and proliferation of cancerous cells. Therefore, melatonin can interfere in the metabolic reprogramming of tumors cells and hence hinder the growth and development of a tumor [42,55].

Besides its direct impact on the metabolism of cancerous cells, melatonin also affects tumor immunity by modulating the tumor microenvironment. For example, inhibition of glutamine metabolism facilitates tumor-specific immunity partly by affecting suppressive myeloid cells, which are integral elements of the tumor microenvironment. This outlines its potential as a versatile therapeutic agent in treating cancers due to its dual action, direct action on cancer cell metabolism, and indirect action through immune modulation [56].

The mechanism of melatonin was also examined in ovarian cancer cells (SKOV-3 and CAISMOV-24) focusing on energy metabolism and kinase signaling. The cells were segregated into control and melatonin-treated groups, with or without the antagonist luzindole. The treatment showed reduced levels of HIF-1α, G6PDH, GAPDH, PDH, and CS, regardless of luzindole’s presence. Moreover, melatonin decreased the activity of significant OC-related enzymes such as PFK-1, G6PDH, LDH, CS, and GS, but increased PDH activity. Lactate and glutamine levels were also reduced following melatonin exposure. Furthermore, melatonin decreased the oncogenic signaling molecules such as CREB, JNK, NF-κB, *p*-38, ERK1/2, Akt, P70S6K, and STAT in both cell lines. These findings demonstrate that melatonin suppresses Warburg-type metabolism and potentially represses glutaminolysis, thereby suppressing the activation of oncogenic pathways during ovarian cancer progression and invasion [57].

**Figure 5:**
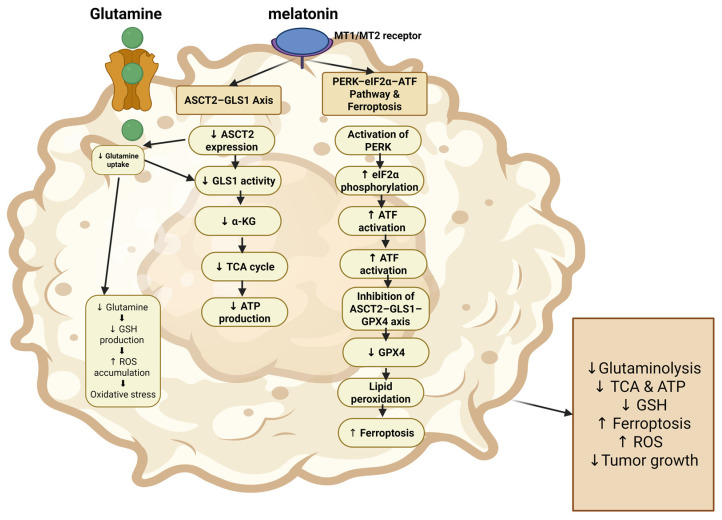
Melatonin is transported into cancer cells and metabolized to produce glutamate then α-ketoglutarate which in turn enter the TCA cycle to contribute to ATP production. Melatonin interferes with this process causing increased lipid peroxidation, reactive oxygen species accumulation, and ferroptosis induction, ultimately suppressing tumor growth. Arrows pointed to the top mean activations, and arrows pointed down mean inhibition.

Another study investigated the effect of melatonin on pancreatic cancer cells by targeting glutamine metabolism. In this study, melatonin inhibited the glutamine metabolism pathway, increased cellular oxidative stress, induced ferroptosis, and exerted anticancer effects. The study showed that melatonin activates the PERK-eIF2α-ATF ER stress pathway, exerting its antitumor effects by inhibiting glutamine metabolism and inducing ferroptosis through the ASCT2-GLS1-GPX4 axis [52]. ASCT2 has been reported as a glutamine transporter, whereas GLS1 targets the degradation of glutamine, contributing to GSH production and a reduction in oxidative stress (Figure 5) [58]. Furthermore, previous studies showed that targeting ASCT2-mediated glutamine metabolism disrupts cellular redox balance, thereby inhibiting the proliferation of pancreatic cells [59].

### 4.3. Targeting Fatty Acid Metabolism

Melatonin exerts anticancer activity by blocking the uptake and metabolism of linoleic acid (omega-6 fatty acid), which is converted into 13-hydroxyoctadecadienoic acid (13-HODE), a mitogenic signaling molecule resulting in the stimulation of cancer cell proliferation. This inhibition is mediated by melatonin receptor-dependent mechanisms, resulting in decreased cyclic AMP (cAMP) levels. Reduced cAMP then inhibits fatty acid transport and metabolism in tumor cells, thereby barring the metabolic events that fuel tumor growth [60,61,62].

**Figure 6:**
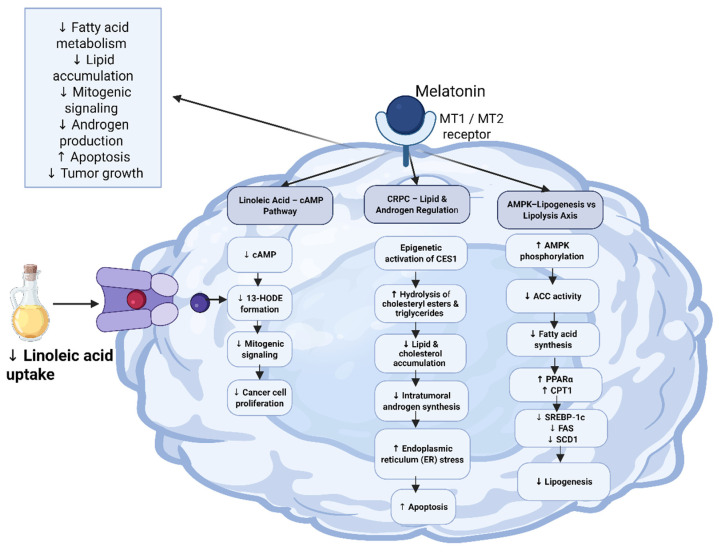
Melatonin targeting fatty acid metabolism through multiple mechanisms including suppressing the uptake of linoleic acid, inhibiting acetyl-CoA carboxylase, and promoting fatty acid oxidation. The net results of melatonin treatment are inhibition of fatty acid-driven mitogenic signaling and apoptosis induction. An upward arrow means increased activity. A downward arrow means decreased activity.

In addition, melatonin has been shown to regulate fatty acid metabolism in CRPC by decreasing lipid and cholesterol accumulation (Figure 6). This effect induces apoptosis by increasing endoplasmic reticulum stress and reducing de novo intratumoral androgen synthesis. These actions are mediated by the epigenetic modification of carboxylesterase 1 (CES1), an enzyme that plays an important role in the hydrolysis of cholesteryl esters and triglycerides [63].

Another study showed that pretreatment with melatonin significantly reduced triglyceride and cholesterol accumulation induced by oleic acid in HepG2 cells. This effect was mediated through phosphorylation of AMP-activated protein kinase AMPK and acetyl-CoA carboxylase ACC, resulting in their activation and inactivation, respectively. Also, melatonin upregulated the expression of peroxisome proliferator-activated receptor-α (PPARα) and its target gene carnitine palmitoyl-CoA transferase 1 (CPT1), which are associated with lipolysis. In contrast, melatonin downregulated sterol regulatory element binding protein-1c (SREBP-1c), fatty acid synthase (FAS), and stearoyl-CoA desaturase-1 (SCD1), which are associated with lipogenesis [64].

### 4.4. Melatonin’s Influence on the Tumor Microenvironment and Metabolic Interactions

Melatonin’s ability to decrease tumor growth has been shown in several forms of cancer such as breast, prostate, colorectal and head and neck cancers. In MCF-7 human breast cancer xenografts, melatonin administration produced a significant reduction in glucose uptake, lactate effluent, and lactic acid metabolism, each of which played a role in decreased tumor cell proliferation [60,62]. Furthermore, in colorectal and cervical cancer models, melatonin antagonized aerobic glycolysis and signaling of fatty acid metabolism, further suggesting melatonin as a potential therapeutic agent for cancer treatment [65].

As melatonin is a modulator of key metabolic pathways in cancer cells, it shows potential as a chronotherapeutic strategy for cancer therapy and prevention. Notably, even at low doses, melatonin is effective; thus, over-the-counter supplements may serve as a workable and accessible intervention in cancer treatment [60,65].

Beyond its metabolic effects, melatonin exerts anticancer effects by regulating cell survival and death pathways. In vitro studies on MCF-7 and MDA-MB-231 breast cancer cell lines have shown that melatonin induces apoptosis by regulating pro- and anti-apoptotic proteins [66]. Melatonin treatment increase intracellular ROS leading to deacetylation of p53 via SIRT1, inhibition of its nuclear translocation and induction of transcription-independent apoptosis [67,68]. Additionally, melatonin downregulates NF-κB and upregulates *p*-JNK which is associated with ROS accumulation and tumor cell apoptosis [69].

**Figure 7:**
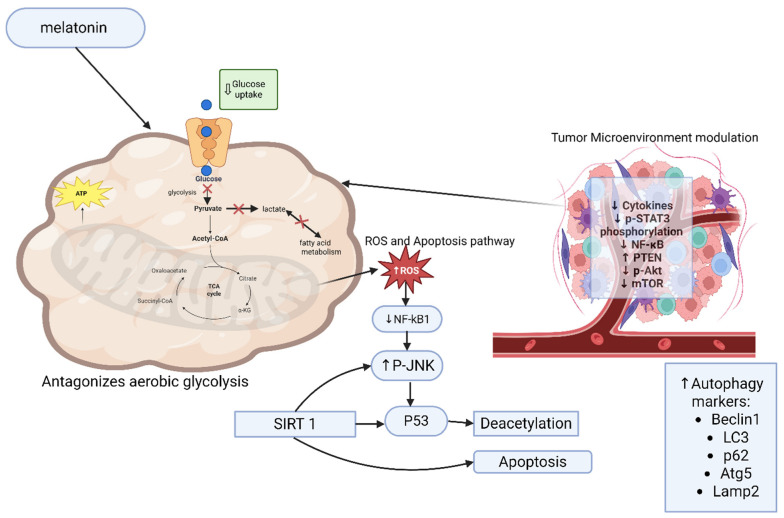
Melatonin’s influence on the tumor microenvironment and metabolic interactions. Melatonin alters mitochondrial metabolism and increases reactive oxygen species in cancer cells by reducing glucose uptake and inhibiting aerobic glycolysis. Induction of apoptosis is achieved through multiple signaling mechanisms including NF-κB inhibition, JNK activation, and SIRT1-mediated p53 deacetylation. Tumor microenvironment is also modulated by melatonin through inflammatory cytokine reduction and targeting other signaling molecules including *p*-STAT3, NF-κB, Akt, and mTOR. Arrows pointed to the top mean activations, arrows pointed down mean inhibition, and arrows with the symbol × mean blocking.

Melatonin also modulates the tumor microenvironment by reducing the production of inflammatory cytokines, thereby inhibiting *p*-STAT3 phosphorylation and nuclear translocation of *p*-STAT3 as well as reducing NF-κB. Furthermore, melatonin regulates various autophagy factors including Beclin-1, p62, ATG5, LAMP2 and LC3. It also increases PTEN, decreases phosphorylated Akt and mTOR and activates autophagy [69]. In addition to its direct metabolic effects, melatonin also regulates key signaling pathways involved in cell survival and apoptosis, such as p53, NF-κB, and AKT (Figure 7). These pathways are linked to metabolic reprogramming in cancer cells and play an important role in modulating tumor metabolism [18].

## 5. Targeting Cancer Using Phytochemicals

Natural compounds have various beneficial properties that improve health and treat cancer [66,70]. The pharmacological activity of several medications was inspired by naturally synthesized compounds such as alkaloids, flavonoids, and taxanes [71]. The high selectivity, low toxicity, and lower cost of phytochemicals against cancer cells led researchers to think about developing new drugs with fewer side effects and lower resistance [1,72,73,74,75].

The selection of natural compounds discussed in this section is based on their ability to modulate metabolic pathways involved in cancer progression, particularly those related to cell proliferation, apoptosis, and metabolic reprogramming. Moreover, these compounds may act synergistically with melatonin in combined therapeutic approaches. Understanding the changes in cancer cell signaling pathways is an essential step for potentially identifying therapeutic targets. Many natural compounds showed effective activity against those signaling pathways in diverse types of cancer cells [66,70] (Table 1).

## 6. Synergistic Effects of Melatonin and Natural Products on Tumor Metabolism

Naturally derived products are known to be an essential component of antitumor therapies. Alkaloids, polyphenolic compounds, and other metabolites have multifaceted effects on cancer cells by regulating altered metabolic energy processes that support tumor growth [86] (Table 2).

Resveratrol combined with melatonin decreased the number of active breast cancer cells and cancer invasion significantly. The antioxidant activity of both compounds inhibited cancerous cell growth and exerted antimutagenic effects [87]. In addition, the codelivery of melatonin and resveratrol via a sericin-based nanocarrier (MR-SNC) showed synergistic cytotoxic effects in MCF-7 breast cancer cells [88]. These effects are associated with metabolic suppression of tumor cells and increased metabolic stress.

Another study compared the effects of curcumin and melatonin administered individually or in combination. The combination of these two agents inhibited bladder cancer cell proliferation more effectively than either compound alone. Melatonin synergizes with curcumin anticancer activity by stimulating the cytochrome *c*/caspase-dependent apoptotic pathway and suppressing IKKβ activity, which leads to the inhibition of the NF-κB/COX-2 signaling pathway [89]. This synergistic effect is linked to the suppression of cancer cell metabolism through inhibition of survival pathways. In light of lycopene and melatonin antioxidant properties, it has been shown that daily supplementation of both can reduce tumor growth [90].

A prior study investigated the ability of the combination of MLT and Andro to inhibit xenografted CRC cells in mice. Consistent with the results in cell lines and mice xenografts, the MLT and Andro combination significantly decreased the number and size of patient-derived tumor organoids compared to treatment with these compounds individually. In summary, their data reveals a potent and synergistic therapeutic effect of MLT and Andro in the treatment of CRC. The dual activation of autophagy by these two anticancer compounds can effectively trigger CRC cell death and arrest tumor development. The results strongly suggest that targeting autophagy-related genes in combination could serve as a potential therapeutic strategy in CRC, either on its own or in conjunction with conventional chemotherapy [86].

In another study, the combination of melatonin and irinotecan with natural compounds such as wogonin and celastrol enhanced cytotoxic activity in colon cancer cells. These effects appear to disrupt the metabolic homeostasis of cancer cells, thereby reducing proliferation and increasing sensitivity in both drug-sensitive and cancer stem-like cells [91].

Moreover, the combination of thymoquinone and melatonin proved to be more potent in inducing an antiproliferative response against the tested cell lines as compared with single treatment. The combination exerted its effect mainly by induction of apoptosis, inhibition of angiogenesis, and shifting the immune response toward the Th1 anticancer response, which are collectively linked to increased metabolic stress and impaired tumor growth [7].

**Figure 8:**
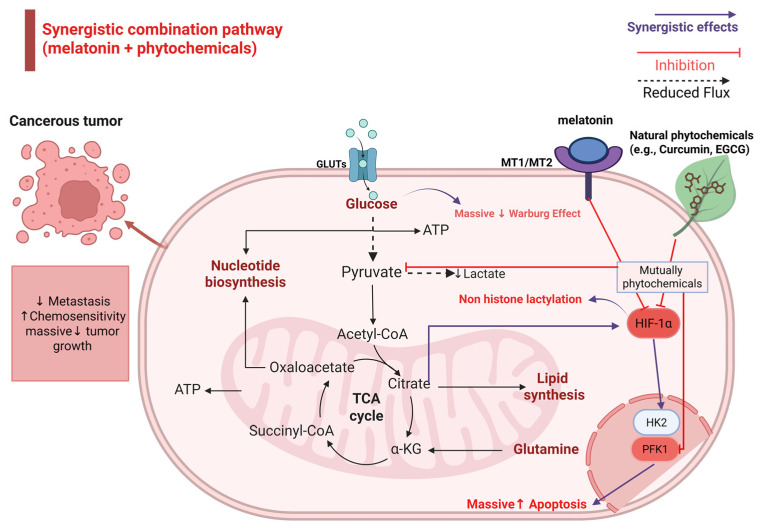
Synergistic potential of melatonin with other natural phytochemicals in regulating tumor metabolism. The synergistic combination triggers a multi-targeted suppression of metabolic reprogramming. Natural phytochemicals complement melatonin’s action by robustly inhibiting the PI3K/Akt/mTOR pathway, leading to a profound downregulation of HIF-1α. This convergence results in the massive inhibition of glucose transporters (GLUTs) and key glycolytic enzymes, including hexokinase 2 (HK2) and pyruvate dehydrogenase kinase 1 (PDK1). Consequently, this metabolic blockade leads to a dramatic reduction in tumor growth and metastasis, while significantly enhancing chemosensitivity and mitochondrial-mediated apoptosis. Upward arrows indicate activation, and downward arrows indicate inhibition.

Studies investigating the combination of melatonin with EGCG have demonstrated synergistic anticancer effects. A significant reduction in COX-2 and HO-1 was observed when it was combined with melatonin. Additionally, melatonin enhanced the ability of EGCG to inhibit Bcl-2 and NF-κB, resulting in increased cytotoxicity. Furthermore, EGCG prevented melatonin-induced activation of Pl3K and COX-2, and melatonin likely sensitized HepG2 cells to EGCG-induced cytotoxicity through downregulation of p21 [92]. These molecular changes contribute to metabolic suppression of tumor growth through reduced energy production and enhanced mitochondrial apoptosis (Figure 8).

Similarly, studies on the combination of melatonin with berberine have shown antitumor activity. For instance, ref. [93] showed that treatment with melatonin effectively enhanced berberine-mediated inhibition of cell proliferation, cell migration and colony formation. Melatonin increased the antitumor activity of berberine by activating caspase/Cyto C and inhibiting AP-2β/hTERT, NF-κB/COX-2 and Akt/ERK signaling pathways. These effects disrupt cancer cell metabolic adaptation, leading to reduced proliferation, migration, and colony formation.

The classification of synergistic effects in Table 2 is based on the strength of available experimental evidence. “Strong synergy” refers to combinations supported by multiple studies showing consistent effects. “Confirmed synergy” indicates experimentally validated interactions, whereas “promising synergy” refers to preliminary evidence. “Hypothetical synergy” represents proposed mechanisms that require further experimental validation.

The reported synergistic effects were mainly based on findings from both in vitro and in vivo studies conducted under different experimental conditions. These include differences in compound concentrations, treatment durations, and administration methods. In most studies, melatonin was used at pharmacological doses ranging from nanomolar to micromolar concentrations, either alone or in combination with plant-derived compounds [45,94]. However, due to heterogeneity among studies, direct comparison of experimental conditions remains limited.

**Table 2:** Bioactive compounds that combine with melatonin to target tumor cells and their mechanism.

Bioactive Compounds	Key Metabolic Targets	Melatonin Synergy	Mechanisms of Synergy	Observed Outcomes	Reference
**Curcumin**	Cytochrome c, IKKß, NF-κB, COX-2,MMP-2, MMP-9, TIMP-2	Strong synergy	- Inhibition of NF-κB signaling. - Inhibition of IKKß activity and NF-κB binding to COX-2 promoter. - Inhibition of MMP-2/9 and activation of TIMP-2.	- Reduced invasive enhanced apoptosis. - Tumor growth inhibition.	[89]
**Resveratrol**	- Apoptosis pathway. - DNA fragmentation. - Chromatin condensation.	Strong synergy	- Antioxidant activity. - Induction of apoptosis. - Induction of DNA damage.	- Enhanced DNA damage & cell death in MCF-7 cells. - Increased cytotoxicity.	[88]
**Thymoquinone**	VEGF, INF, T helper 1	Strong synergy	- Induction of apoptosis and necrosis. - Inhibition of VEGF expression. - Activation of T helper 1 anticancer immune response.	- Reduction in tumor growth. - Enhanced apoptosis and necrosis. - Increased IFN level.	[7]
**Andrographolide**	NR4A1, CTSL, Atg12 Apoptosis	Strong synergy	- Reduction in colony formation. - Induction of apoptosis. - Activation of autophagy.	- Tumor growth inhibition. - Increased apoptosis. - Reduced colony formation.	[86]
**EGCG**	NF-κB, Pl3K, COX-2, HO-1 p21, Bcl-2	Confirmed synergy	- Induction of apoptosis. - Inhibition of NF-κB and Bcl-2. - Downregulation of p21. - Inhibition of Pl3K and COX-2.	- Increased apoptosis, reduced migration, enhanced cytotoxicity.	[92]
**Wogonin**	- Apoptosis induction. - Migration inhibition. - Elimination of CSC-like cells.	Promising synergy	- Induction of apoptosis. - Inhibition of cell proliferation and migration.	- Reduced tumor growth. - Inhibition of migration.	[91]
**Berberine**	Caspase-9 cytochrome c Bcl2, AP-2ß NF-κB, COX-2, Akt/ERK	Confirmed synergy	- Activation of caspse-9/Cyto C - Inhibition of AP-2ß/hTERT,NF-kß/COX-2 and Akt/ERK signaling pathways.	- Reduced proliferation, colony formation and cell migration.	[93]
**Quercetin**	HK2, MAPK, Akt/mTOR,	Hypothetical synergy	- Inhibition of HK2 & Akt/mTOR pathways.	- Preclinical evidence only, synergy not yet tested with melatonin.	[95]
**Matrine**	HIF-1α, GLUT1, HK2, LDHA	Hypothetical synergy	- Inhibition of HIF-1α signaling. - Suppression of glycolysis.	- Needs empirical validation.	[96]
**Chrysin**	HK2 (mainly), NF-κB	Hypothetical synergy	- Inhibition of NF-κB signaling. - Suppression of glycolysis.	- No direct synergy data yet.	[97]
**Baicalin**	Bcl-2, Caspase 3 and -9 MMP2; MMP 9	Hypothetical synergy	- Induction of apoptosis. - Inhibition of migration.	- Unverified combinations.	[98]

## 7. Future Perspective

Current evidence shows that melatonin, in combination with natural compounds, has anticancer effects [94]. However, most available studies are still preclinical, and there is a lack of well-designed clinical trials to confirm their effectiveness in cancer patients. Further clinical studies are needed, with an emphasis on developing improved drug delivery systems, such as nanoparticle-based approaches, to enhance the therapeutic potential of these compounds. In addition, more research is needed to better understand the metabolic and molecular mechanisms underlying these synergistic effects, particularly their role in cancer metabolism.

## 8. Conclusions

An extremely attractive development in the field of oncology is represented by the combination of melatonin and phytochemicals. This review has discussed diversified mechanisms for anticancer action: modulating cancer metabolism, inducing apoptosis, inhibiting angiogenesis, and synergism with other natural compounds. Such a combination enhances the potential of phytochemicals as a complementary approach to conventional cancer therapies, with reduced toxicity and improved patient outcomes. The importance of this review article stems from the fact that it presents multiple opportunities for researchers to conduct experimental and clinical research focusing on refining the combinations of melatonin and phytochemicals, evaluating their efficacy in cancer research, and exploring their mechanisms in greater depth. This approach has pointed out the possible use of compounds of natural origin in experimental cancer research.

## Figures and Tables

**Table 1:** Anticancer activity of some bioactive compounds.

Bioactive Compounds	Source	Type of Cancer	Mechanism	Reference
**Honokiol**	*Magnolia officinalis*	MCF7 breast, ovarian RKO colon, LNCaP prostate	- Inhibition of RAS signaling. - Induction of cyclophilin D expression. - Activation of mitochondrial transition pore.	[76]
**Curcumin**	Turmeric	HCT116 colon, gastric, breast, lung	- Interference with G1/M cell growth phase. - Induction of apoptosis. - Inhibition of cyclin D1 expression.	[77,78]
**Resveratrol**	Grapes	Colon, lung, breast, oral, prostate	- Scavenging of reactive oxygen species. - Inhibition of MMP-9. - Disruption of mitochondrial membrane potential.	[79,80]
**Ursolic acid** **Koetjapic acid**	Apple, prunes, and pears	Colon	- Inhibition of STAT3 signaling. - Induction of caspase-3-mediated apoptosis.	[81]
**Paclitaxel**	*Taxus brevifolia* (Pacific yew tree)	Metastatic breast cancer, ovarian cancer	- Stabilization of microtubules. - Inhibition of mitosis (G2/M cell cycle arrest). - Induction of apoptosis.	[82]
**Lycopene**	Red fruits and vegetables	Breast (MCF-7, MDA-MB-231), colon, prostate	- Inhibitino of glycogen synthase kinase-3β. - Inhibition of β-catenin signaling.	[83]
**Licochalcone A**	*Glycyrrhiza glabra*	Breast	- Induction of apoptosis. - Inhibition of cell proliferation.	[82]
**Gingerol**	*Zingiber officinale*	Colon (HCT116), cervical (HeLa), liver (HepG2, Huh7), breast (MCF7)	- Antioxidant activity. - Synergistic activity with doxorubicin.	[84]
**Emodin**	*Aloe vera*	Lung cancer (A549 cells)	- Inhibition of protein kinase. - Inhibition of p53 protein aggregation.	[85]

## Data Availability

The data presented in this study are available on request from the corresponding author.

## Abbreviations

**Abbreviation**	**Full Meaning**
ACAD	Acyl-CoA Dehydrogenase
ACC	Acetyl-CoA Carboxylase
ACLY	ATP-Citrate Lyase
AGPAT	Acylglycerol-3-Phosphate Acyltransferase
AKT	Protein Kinase B
AMPK	AMP-Activated Protein Kinase
ASCT2	Alanine-Serine-Cysteine Transporter 2
ATP	Adenosine Triphosphate
Bcl-2	B-cell Lymphoma 2
CES1	Carboxylesterase 1
COX-2	Cyclooxygenase-2
CPT1	Carnitine Palmitoyltransferase 1
CREB	cAMP Response Element-Binding Protein
CS	Citrate Synthase
CSC	Cancer Stem Cell
DNL	De Novo Lipogenesis
EGCG	Epigallocatechin Gallate
ER	Estrogen Receptor
ERK	Extracellular Signal-Regulated Kinase
FASN	Fatty Acid Synthase
FH	Fumarate Hydratase
G6PDH	Glucose-6-Phosphate Dehydrogenase
GAPDH	Glyceraldehyde-3-Phosphate Dehydrogenase
GDH	Glutamate Dehydrogenase
GLS	Glutaminase
GLS1	Glutaminase 1
GLUT1	Glucose Transporter 1
GPX4	Glutathione Peroxidase 4
GSH	Glutathione
HIF-1α	Hypoxia-Inducible Factor 1 Alpha
HK2	Hexokinase 2
HO-1	Heme Oxygenase 1
IFN	Interferon
IKKβ	IκB Kinase Beta
JNK	c-Jun *N*-terminal Kinase
LC3	Microtubule-Associated Protein 1A/1B-Light Chain 3
LDH	Lactate Dehydrogenase
LDHA	Lactate Dehydrogenase A
LPL	Lipoprotein Lipase
MAPK	Mitogen-Activated Protein Kinase
MCT4	Monocarboxylate Transporter 4
MLT	Melatonin
MMP-2	Matrix Metalloproteinase 2
MMP-9	Matrix Metalloproteinase 9
NF-κB	Nuclear Factor Kappa B
NR4A1	Nuclear Receptor Subfamily 4 Group A Member 1
OC	Ovarian Cancer
OXPHOS	Oxidative Phosphorylation
PDH	Pyruvate Dehydrogenase
PERK	Protein Kinase RNA-like Endoplasmic Reticulum Kinase
PFK-1	Phosphofructokinase-1
PI3K	Phosphoinositide 3-Kinase
PKM2	Pyruvate Kinase M2
PPARα	Peroxisome Proliferator-Activated Receptor Alpha
PTEN	Phosphatase and Tensin Homolog
ROS	Reactive Oxygen Species
SCD1	Stearoyl-CoA Desaturase 1
SCN	Suprachiasmatic Nuclei
SDH	Succinate Dehydrogenase
SIRT1	Sirtuin 1
SLC1A5	Solute Carrier Family 1 Member 5
SREBP-1c	Sterol Regulatory Element-Binding Protein 1c
STAT3	Signal Transducer and Activator of Transcription 3
TAG	Triacylglycerol
TCA	Tricarboxylic Acid Cycle
TIMP-2	Tissue Inhibitor of Metalloproteinases 2
Th1	T Helper Type 1
VEGF	Vascular Endothelial Growth Factor
cAMP	Cyclic Adenosine Monophosphate
mTOR	Mammalian Target of Rapamycin
β-oxidation	Fatty Acid Beta Oxidation
